# Muscle Deflection Used as an Assessment Indicator of the Rehabilitation Quality After Total Hip Joint Arthroplasty (THA)

**DOI:** 10.3390/jcm14113728

**Published:** 2025-05-26

**Authors:** Radu Vișan, Marjan Mihajlov, Malaete Alina Luminița, Alexandra Irma Gabriela Baușic

**Affiliations:** 1 Department of Ortopedics and Traumatology, “Carol Davila” University of Medicine and Pharmacy, 020021 Bucharest, Romania; 2 Floreasca Emergency Clinical Hospital, 014461 Bucharest, Romania; 3 Enayati Medical City, 013812 Bucharest, Romania; alinamalaete@yahoo.com; 4 Department of Obstetrics and Gynecology, “Carol Davila” University of Medicine and Pharmacy, 020021 Bucharest, Romania; alexandra.bausic@drd.umfcd.ro

**Keywords:** AROM, PROM, muscle deflection, abduction, extension, flexion

## Abstract

**Background/Objectives:** Total hip joint arthroplasty (THA) is a common procedure aimed at alleviating pain and restoring mobility in patients with severe hip joint conditions, particularly osteoarthritis. While the surgery itself is effective, postoperative rehabilitation is crucial for long-term functional recovery. This study investigates the role of muscle deflection as an indicator of rehabilitation quality, focusing on hip movement improvements (abduction, extension, and flexion) over an 8-week recovery period. The objective is to assess whether muscle deflection measurements can serve as reliable metrics for evaluating recovery progress and functional outcomes. **Methods**: The study involved post-THA patients from Euroclinic Hospital S.A. and Bucharest Emergency Clinical Hospital, who were divided into an experimental group (undergoing a specialized rehabilitation program) and a control group (receiving standard recovery protocols). Measurements of active range of motion (AROM), passive range of motion (PROM), and muscle deflection were taken using a handheld digital dynamometer (ActivForce 2) at different time points (2, 4, 6, and 8 weeks post-surgery). The Western Ontario and McMaster Universities Osteoarthritis Index (WOMAC) was also used to assess pain, physical difficulties, and joint stiffness. **Results**: The study found progressive improvements in AROM, PROM, and muscle deflection across all movements over the 8-week period. **Conclusions**: The findings highlight the importance of structured physiotherapy in post-THA rehabilitation. The use of muscle deflection measurements provides an objective and quantifiable method for tracking patient progress. Future rehabilitation protocols could benefit from incorporating muscle deflection monitoring to tailor recovery programs and optimize patient outcomes. Standardizing rehabilitation exercises, including balance training and resistance exercises, may further enhance recovery and reduce long-term functional impairments.

## 1. Introduction

When hip discomfort makes it difficult for a person to walk or bend over to put on their shoes or socks, it has a major negative influence on their quality of life. Among musculoskeletal conditions, diseases of the hip joint are recognized as important causes of pain and functional impairment, surpassed in frequency by disorders of the lower back [1]. Hip arthroplasty is the sole option to ease the pain and resume the comforts of a regular life if the recommended medication and/or the employing of a frame, crutches, or cane are insufficient to relieve the pain.

A hip prosthesis is used to replace the injured coxo-femoral joint during the surgical operation known as arthroplasty, sometimes called a hip replacement [2]. The total hip prosthesis (which joins the two previously mentioned components) is composed of the head of the prosthesis; the stem or tail of the prosthesis, which is put into the femur; and the cup, which sits at the level of the pelvis [2]. Anatomically speaking, the joint is a system composed of both passive and active components. Since the joint is a moveable structure, the ligaments have a mechanical function in controlling the joint’s range of motion, guiding the contact surface, and strengthening the joint [2].

The hip joint (coxo-femoral), a synovial joint (acetabular cavity), connects the femur bone (femoral head) to the pelvic skeleton [3]. In 1940, Dr. Austin Moore carried out the first hip arthroplasty, or hip replacement [4]. After significant improvements in surgical technique, implant complexity, and material quality over the past 70 years, hip arthroplasty is currently thought to be the most successful orthopedic treatment [4]. According to Sloan et al., the number of hip arthroplasties performed in the US is expected to rise by 71% between 2018 and 2030, or over 635,000 treatments [5].

In most nations, the frequency of hip and knee replacements has dramatically grown since 2000 [5]. Germany, Austria, Switzerland, Finland, Luxembourg, and Belgium had some of the highest rates of hip and knee replacement surgery in 2017 [6]. In the United States, an estimated 170,000 hip arthroplasties were carried out year [7]. One in eight women and one in ten males in Australia have had an arthroplasty (ATS) [7,8,9]. Israel, Ireland, and Korea have the lowest rates [10]. Between 2007 and 2017, hip replacement rates generally rose by 30% [6].

It is noteworthy that while the frequency of ATS has increased, the average age of patients in need of this kind of intervention has decreased. The age group that underwent the surgical operation the most commonly in the United States between 2001 and 2007 was 20 to 49 years old [11]. The main objectives of total hip replacement are pain relief and joint mobility restoration [11]. If there are no postoperative complications and the patient receives the appropriate amount of rehabilitation treatment, the functional prognosis for total arthroplasty patients is excellent [12]. More study is still needed on recovery in ATS, including when to use it (pre- and/or postoperatively) as well as how long recovery programs should last and be structured [12].

Up to two years after total hip replacement, patients may also have functional impairments (decreased muscular strength, postural stability, or walking speed) that might impact the duration and kind of rehabilitation [13,14,15].

Poor recovery and postoperative care may be associated with a higher risk of falls, ipsilateral hip OA development, and prosthesis displacement [15]. One of the primary reasons for total hip joint arthroplasty (THA) is osteoarthritis [13,14,15].

However, following surgery, certain functional and unpleasant limitations could last for a very long period. Because of this, the THA surgical method alone is not enough; rehabilitation exercises should be used for a long time. Hip replacement surgery is insufficient if a physical therapist does not recommend a suitable medical recovery.

Physical therapy and rehabilitation are essential for medical recovery after surgery. They start immediately after the operation, continue at home and in the hospital, and may be necessary for up to a year after the patient is discharged. It is impossible to accurately forecast how long the medical recuperation will take. Depending on each instance, the kind of operation (total or partial), and any comorbid conditions, the physical therapist can estimate the time needed for a full recovery. As a result, following total hip replacement surgery, a physiotherapist must prescribe rehabilitation activities [4].

### New Approaches to the Relationship Between Surgical Intervention and Recovery

The main goals of recovery are to regain hip stabilizer muscle strength and joint mobility [16]. The re-education of balance and proprioceptive deficits, however, appears to play a significant role in recovering physiological function and quality of life. Accordingly, Labanca et al. emphasize that arthrosis and major joint replacement surgery can damage some joint structures and surrounding elements, with the mechanoceptors in the joints being the elements most impacted by surgery in ATS [17].

Proprioception is impaired by damage to mechanoceptors. Proprioceptive signals that are abnormal have an impact on both sensory and motor functions since sensory information is necessary for movement programming [18]. These factors account for patients’ sensory and motor deficits following joint replacement surgery [19,20].

The biomechanics of functional motions may also be impacted by proprioception abnormalities. It is not surprising that people with ATS still experience gait impairments a year following surgery [18]. Hip proprioception may be impacted in various ways depending on the surgical technique used to treat ATS. In reality, other treatments, such the lateral and posterior techniques, can also influence the muscles and tendons, inflicting more extensive harm than the direct anterior approach (DAA), which only affects the hip joint capsule substantial proprioceptors [21]. The influence of different surgical methods on hip biomechanics and clinical results has been widely explored [22,23], although proprioception abnormalities in this setting have not yet been adequately studied. Because there is not much research on the benefits of balance training after ATS that have been published in specialized meta-analyses, it is also unclear what kinds of exercises should be performed and how they ought to be distinguished in different periods of recovery following ATS surgery [24].

The preoperative health status of the patients, including their level of muscle strength, is considered to be a factor associated with favorable postoperative outcomes following total joint replacement, based on an analysis conducted in 2021 by Sauersig and colleagues of 26 individual investigations with 1004 participants and 32 randomized clinical trials involving 1753 individuals [25,26].

The stage of functional recovery that patients must go through following surgery, with the distinction of an early phase and a late phase of recovery, is the subject of rich literature in contrast to this aspect. Di Monaco et al. conducted a meta-analysis on the efficacy of exercise programs after ATS and found that the most often used exercise regimens in the early postoperative period are neither endorsed nor refuted by controlled clinical studies [27].

Other studies show that early progressive application and higher-intensity exercise improve outcomes, patient satisfaction, and adherence to the recovery program and also lessen complications and costs [7,28]. The best outcomes were seen for a 4- to 8-week program of therapeutic activities, with a frequency of two to three times per week, according to an expert agreement on best practices for rehabilitation after ATS [29].

A large segment of the specialized literature is devoted to the specific therapeutic exercises used in functional recovery following hip arthroplasty, as well as to the advantages of recovery and the criteria used to assess these advantages [8,9].

Lowe et al. began reviewing research on the effects of exercise therapy following primary unilateral total hip replacement in terms of enhancing hip joint mobility and range of motion, hip muscle strength, and overall quality of life while also improving functionality. They pointed out that the usefulness of FKT exercises following hip arthroplasty has not been thoroughly examined in numerous systematic reviews [30].

The researchers were drawn to trials in which hip arthroplasty patients also took part in therapeutic exercise recovery intervention programs at various facilities or at home following hospital discharge.

This study aimed to evaluate whether muscle deflection measurements can serve as reliable and objective indicators of rehabilitation quality and functional recovery in patients following total hip arthroplasty over an eight-week postoperative period.

Hypothesis: Patients undergoing a structured rehabilitation program after THA will show significantly greater improvements in muscle deflection, range of motion, and patient-reported functional outcomes compared to those following standard recovery protocols.

## 2. Materials and Methods

Physical activity plays a therapeutic role in hip arthroplasty recovery, and it is important to identify the most effective ways to offer the physiokinetotherapy intervention, in terms of both structure and content.

This covers both psychological and physical effects (exercise types and administration methods). In this case, the variables and instruments specified in Table 1 were used.

Participants in the experimental research were recruited as they were discharged after surgery at Euroclinic Hospital S.A. and the Bucharest Emergency Clinical Hospital. Participants were assigned to one of two groups: an experimental group that consisted of 21 participants, which followed a specialized postoperative rehabilitation program developed by the study’s author, and a control group (20 participants) that received standard postoperative care based on existing hospital protocols.

Eligible participants were recruited upon discharge from surgery at both medical centers. Initial evaluations were conducted on the day of discharge, with follow-up assessments performed at the end of the respective recovery programs, using the instruments detailed in the corresponding section of this paper.

Group assignment was determined based on patient preference at the time of discharge: the experimental group participated in a structured postoperative rehabilitation program based on our methodology, and the control group followed varied conventional rehabilitation routines, depending on standard care practices. Different postoperative recovery regimens were used by the control group.

Candidates needed to be between the ages of 35 and 50, have undergone total hip replacement surgery, and voluntarily participate in the study. Exclusion criteria include a mental health condition, a damaged or diseased hip joint, or significant postoperative complications including hemarthrosis. The exercise program for postoperative recovery begins in the second week after surgery. We aimed to answer these research questions:

1. Can muscle deflection measurements reliably track improvements in hip joint function (abduction, extension, and flexion) after THA?
2. Does a structured rehabilitation program result in greater improvements in muscle deflection and range of motion compared to standard recovery protocols?
3. How do changes in muscle deflection correlate with patient-reported outcomes of pain, stiffness, and physical difficulty following THA?

The rehabilitation program applied to the experimental group was designed to promote functional recovery after THA, focusing on improving joint mobility, muscular strength, proprioception, and gait. The intervention began during the second postoperative week and continued for eight weeks, with three sessions per week, each lasting approximately 60 min.

The protocol included the following components:

1. Passive Techniques (15–20 min per session):
  - Manual therapy: Gentle joint mobilizations and myofascial release targeting periarticular structures (gluteal region, hip flexors, and iliotibial band);
  - Passive range of motion: Performed within pain-free limits to improve joint flexibility and reduce stiffness.
2. Active Therapy (30–35 min per session):
  - Active range of motion: Hip flexion, extension, abduction, and external rotation exercises performed supine or standing, progressing in difficulty;
  - Strengthening exercises: Isometric exercises targeting hip abductors, extensors, and flexors, and progressive resistance training using elastic bands or body weight (e.g., bridges, leg raises).

The control group received standard post-THA care, which varied by institution and included general movement guidelines and unsupervised home exercises but no structured physical therapy program.

Manual muscle testing (MMT) has been used several times to evaluate muscle strength [30]. However, MMT for strength assessment has been criticized for its subjectivity in quantifying muscle force analysis [31].

Hand-held dynamometers (HHDs) are an alternative to MMT for objectively tracking patients’ strength improvement over time. They are capable of reliably measuring muscle force and are useful in muscle deflection analysis [32].

Muscle strength and joint range of motion were assessed using the ActivForce 2 digital dynamometer, a modern handheld dynamometer (HHD) designed for clinical and research applications. This device enables objective quantification of muscular performance and joint mobility through real-time digital feedback and data logging [33]. The ActivForce 2 was utilized to measure the following parameters:

- Peak force: Maximum voluntary contraction during isometric testing;
- Average force: Mean force generated across repetitions or hold duration;
- Force range: Variability between minimum and maximum recorded forces;
- Active range of motion (AROM): The degree of movement initiated and controlled voluntarily by the patient;
- Passive range of motion (PROM): The total range of joint movement achieved with external assistance, without patient muscle activation;
- Bilateral symmetry comparison: Side-to-side comparisons of both strength and range of motion to detect asymmetries between the operated and non-operated limbs [34].

The device was applied according to the manufacturer’s guidelines, with standardized patient positioning and resistance application to ensure consistency across assessments. Measurements were recorded at baseline (day of discharge) and at the conclusion of the rehabilitation program to monitor functional progress and treatment effectiveness [33].

**Figure 1:**
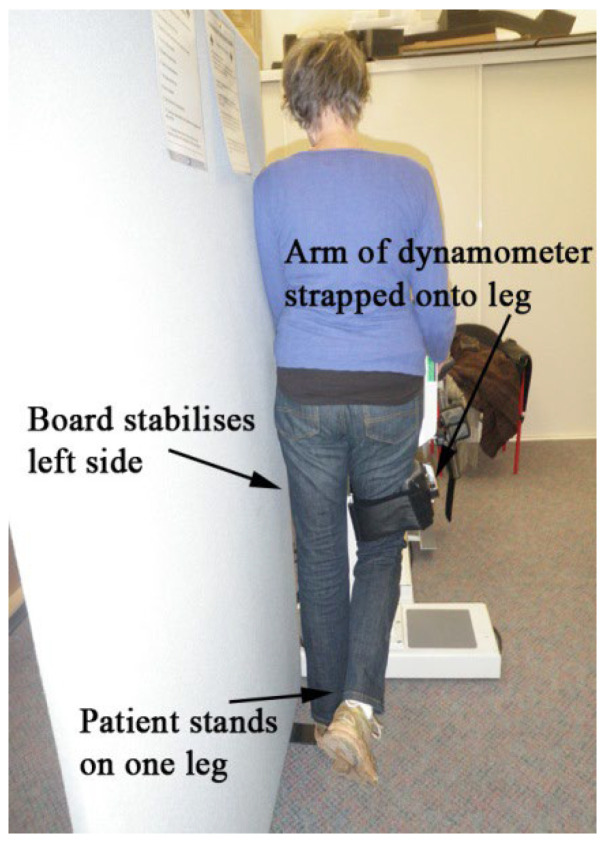
Setup for testing hip abductor muscle deflection.

Written informed permission was given by each participant, who also had their eligibility verified and their rights upheld. The HHD was administered to the patient while standing upright (Figure 1) for 2, 4, 6, and 8 weeks following THA rehabilitation activities. The hip and leg on the other side of the test were pressed against a wall to stabilize that side. The patient’s leg was abducted 10°, and the dynamometer was placed on its lateral epicondyle. In order to keep control of the dynamometer and withstand the patient’s violent abduction, the examiner was braced.

## 3. Results

### Data Acquisitions

Below are the experimental results on deflection in abduction, extension, and flexion obtained 2, 4, 6, and 8 weeks following the THA recovery activities—Table 2, Table 3, Table 4, Table 5, Table 6, Table 7, Table 8, Table 9, Table 10, Table 11, Table 12 and Table 13.

The Table 2, Table 3, Table 4, Table 5, Table 6, Table 7, Table 8, Table 9, Table 10, Table 11, Table 12 and Table 13 summarize the experimental results on deflection in abduction, extension, and flexion obtained 2, 4, 6, and 8 weeks following the THA rehabilitation activities.

Analyzing the tables, we can conclude:

1. In AROM abduction after 8 weeks, deflection increases of 1.61° over the first week are observed.
2. In PROM abduction after 8 weeks, deflection increases of 1.94° over the first week are observed.
3. In both AROM and PROM abduction, the deflection in weeks 6–8 and 4–8 grows and the percentage difference is the same, at 1.02%, which means that a tendency towards normalization appears.
4. In AROM extension after 8 weeks, deflection increases of 2.29° over the first week are observed.
5. In PROM extension after 8 weeks, deflection increases of 2.04° over the first week are observed.
6. In both AROM and PROM extension, the deflection in weeks 6-8 and 4-8 grows and the percentage difference is the same, at 0.42%, which means that a tendency towards normalization appears.
7. In both AROM and PROM extension, the average value of percentage difference is 0.51, which means that a tendency towards normalization appears.
8. In AROM flexion after 8 weeks, deflection increases of 1.79° over the first week are observed.
9. In PROM flexion after 8 weeks, deflection increases of 1.92° over the first week are observed.

The results of the measured deflection can be used as an assessment indicator of the rehabilitation quality after THA. Increased deflection values mean better mobility, a reduction in pain, and an improvement in walking speed.

From this table, AROM, PROM, angle difference, and percentage difference variation depending on time (weeks) were plotted via the Matlab software package, and the following figures were obtained.

A WOMAC test was utilized to confirm the findings of the experimental data acquisition of deflection in abduction, extension, and flexion during a period of 2, 4, 6, and 8 weeks following the THA recovery activities. Patients with osteoarthritis of the knee and hip, including joint pain and physical dysfunction, were assessed using a series of standardized questionnaires by the Western Ontario and McMaster Universities Osteoarthritis Index (WOMAC). On a scale of 0 to 4, the exam questions were rated as follows: None (0), Mild (1), Moderate (2), Severe (3), and Extreme (4). The table below displays the WOMAC test results.

AROM and PROM abduction increased by 1.61° and 1.94° degrees, respectively, compared to the first week. The percentage difference in abduction (for comparison of weeks 6–8 and 4–6) was consistently 1.02%, indicating a trend toward normalization. In extension, both AROM and PROM showed a 0.42% percentage difference, again suggesting normalization tendencies. The average percentage difference between AROM and PROM extension overall was 0.51%, reinforcing the normalization trend. AROM and PROM flexion improved by 1.79° and 1.92° degrees, respectively, after 8 weeks. Regarding patient-reported outcomes, after 8 weeks there was a 37.97% reduction in pain, 32.05% reduction in physical difficulties, and 15.29% reduction in joint stiffness.

Although the observed changes in joint deflection angles (ranging from approximately 1.6° to 2.3°) may appear modest and fall below conventional thresholds for statistical significance due to low variability and limited sample size, they are nonetheless clinically meaningful. In the context of post-THA recovery, even small gains in active and passive range of motion (AROM and PROM) can significantly impact functional mobility, reduce discomfort, and enhance patients’ quality of life. The consistent trend toward normalization—evidenced by parallel percentage improvements in both AROM and PROM (e.g., 1.02% in abduction and 0.42% in extension)—supports a systematic and progressive restoration of joint function. These objective findings are further validated by patient-reported outcomes, with the WOMAC index showing a 37.97% reduction in pain, 32.05% reduction in physical difficulty, and 15.29% decrease in joint stiffness over the 8-week recovery period. Taken together, these results underscore the clinical value of the specialized rehabilitation program, demonstrating that even subtle biomechanical improvements can lead to meaningful functional recovery and enhanced patient satisfaction.

## 4. Discussion

**Figure 2:**
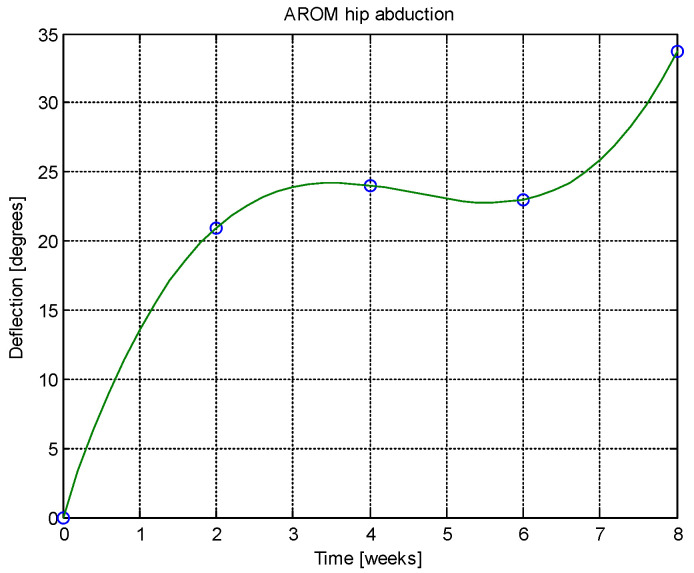
AROM hip abduction versus time.

**Figure 3:**
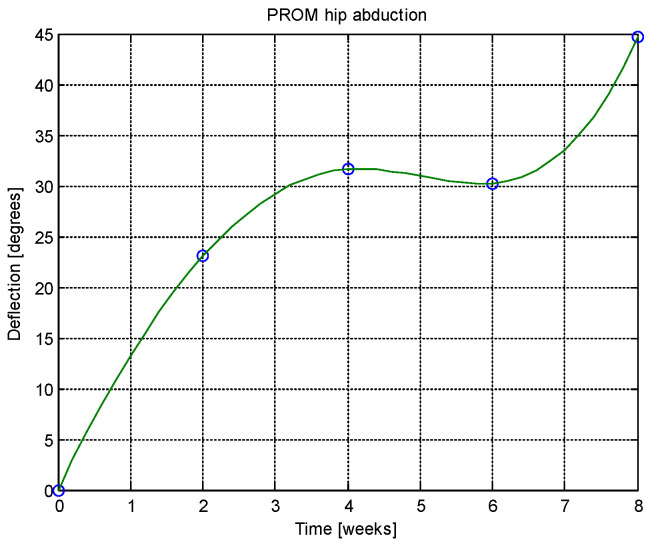
PROM hip abduction versus time.

**Figure 4:**
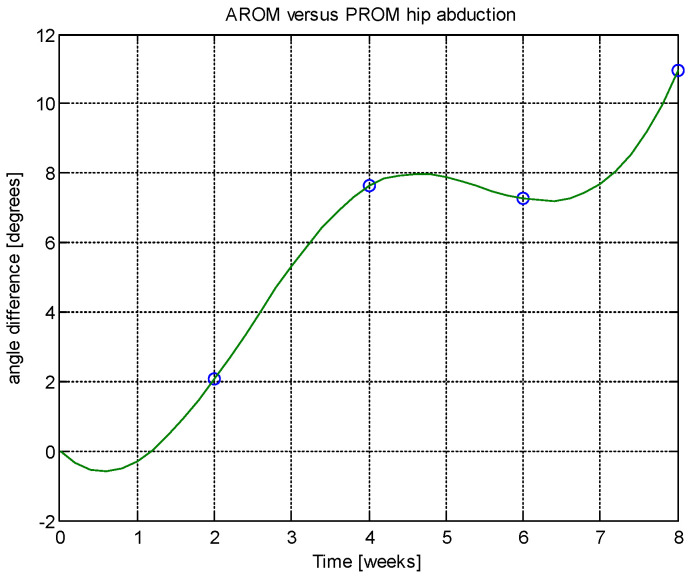
Angle difference between AROM and PROM.

**Figure 5:**
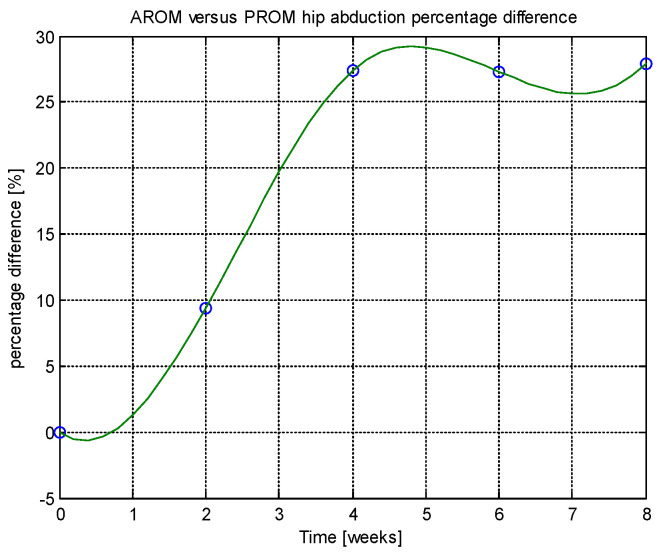
Percentage difference.

**Figure 6:**
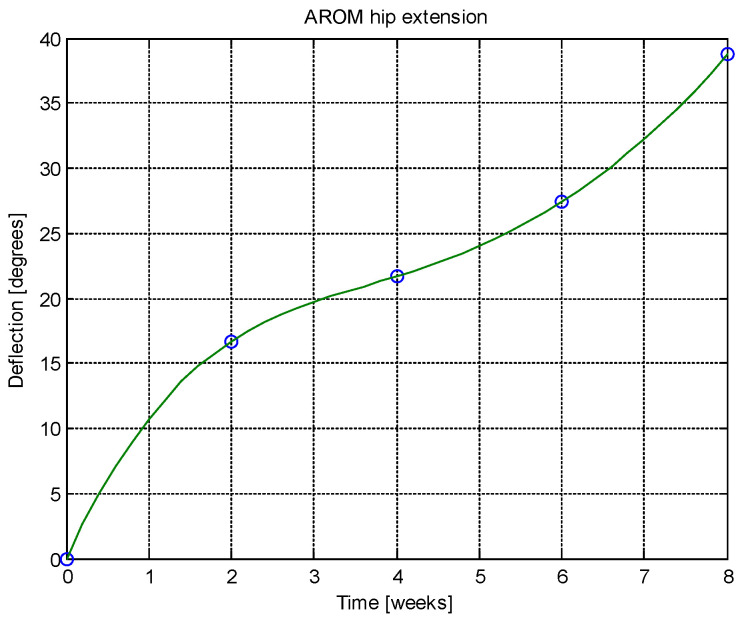
AROM hip extension versus time.

**Figure 7:**
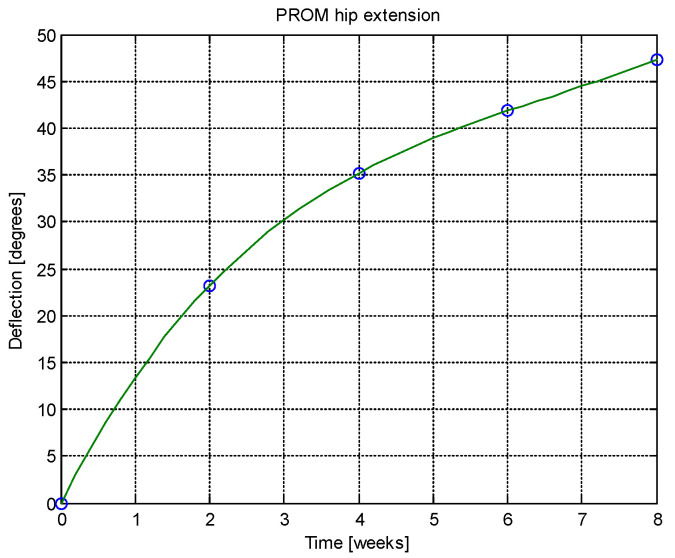
PROM hip extension versus time.

**Figure 8:**
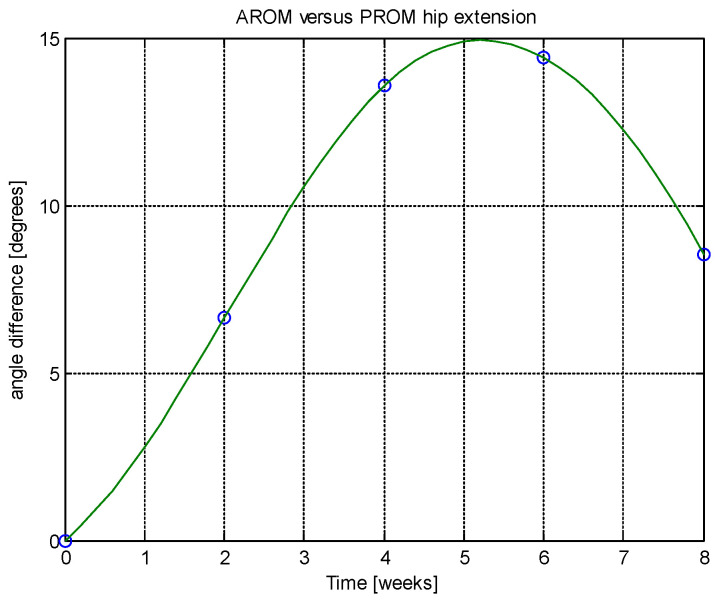
Angle difference between AROM and PROM.

**Figure 9:**
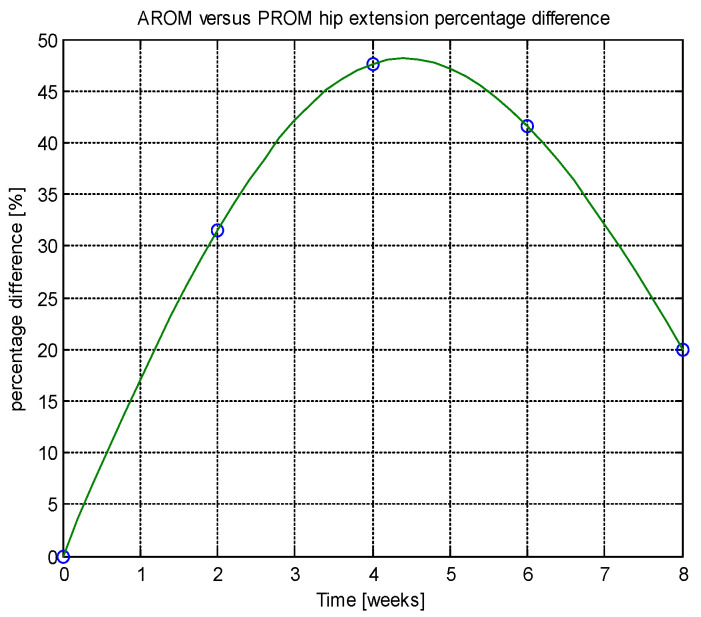
Percentage difference.

**Figure 10:**
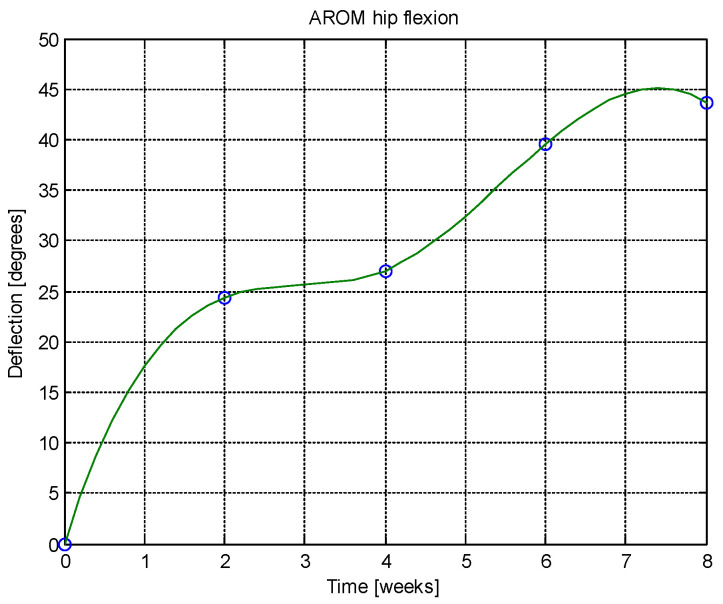
AROM hip abduction versus time.

**Figure 11:**
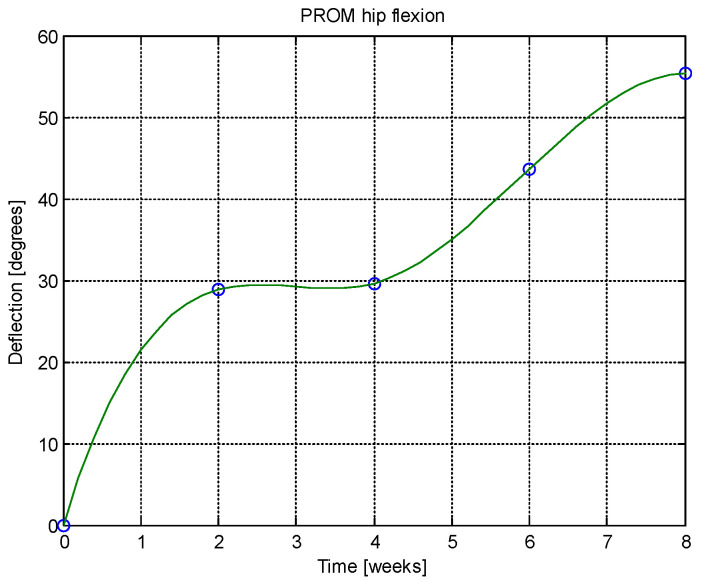
PROM hip abduction versus time.

**Figure 12:**
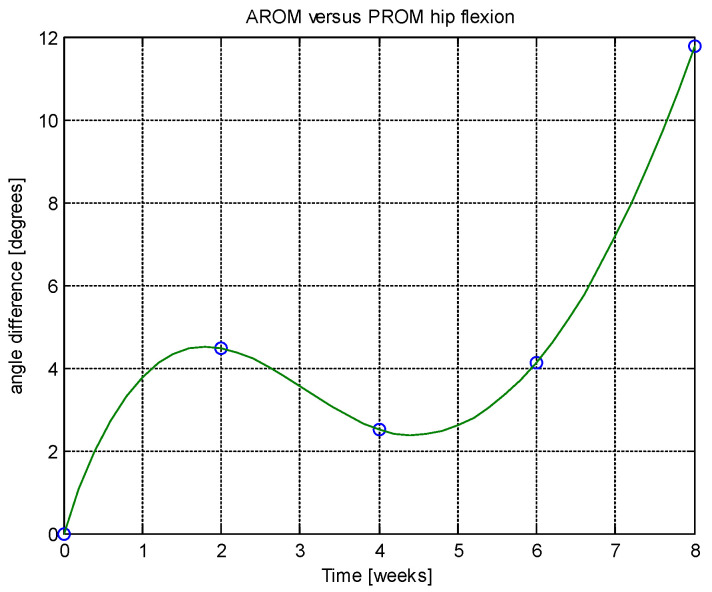
Angle difference between AROM and PROM.

**Figure 13:**
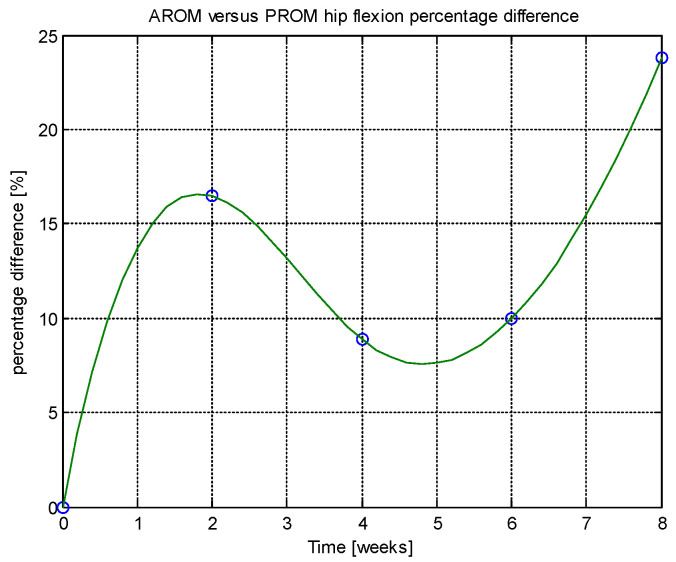
Percentage difference.

Analyzing Table 14 and Table 15 and Figure 2, Figure 3, Figure 4, Figure 5, Figure 6, Figure 7, Figure 8, Figure 9, Figure 10, Figure 11, Figure 12 and Figure 13, we can conclude that the data show progressive and consistent improvements across all measured parameters after 8 weeks of recovery exercises, both objectively (deflection degrees) and subjectively (pain, difficulty, stiffness), with a clear trend toward normalization in joint function. After 8 weeks of recovery exercises, there were significant improvements in both active (AROM) and passive (PROM) range of motion (ROM) for abduction, extension, and flexion movements. The measured deflection can be utilized as a measurement for the effectiveness of rehabilitation following THA. Increased deflection values translate into improved mobility, decreased pain, and faster walking.

Muscle deflection measurements reliably tracked improvements in hip function. Over the eight-week period, there were consistent increases in both active (AROM) and passive (PROM) range of motion across abduction, extension, and flexion, alongside a trend toward normalization. This demonstrates that muscle deflection is a useful and objective indicator of rehabilitation progress following THA.

Although the study compared an experimental group with a structured program to a control group with standard care, the detailed control group outcomes were not provided for direct comparison. However, the significant and progressive improvements observed in the experimental group (especially in mobility gains and reduced pain and stiffness) suggest that structured rehabilitation likely offers superior results over standard protocols.

Changes in muscle deflection were strongly associated with improvements in patient-reported outcomes. After eight weeks, there were notable reductions in pain (37.97%), physical difficulty (32.05%), and joint stiffness (15.29%), according to the WOMAC questionnaire. This correlation supports the use of muscle deflection measurements not only for assessing joint function but also for predicting improvements in overall patient well-being.

A study on the results of a *physiokinetotherapy* program in a trauma unit that was carried out in accordance with standardized care recommendations for a total joint replacement is reported by López-Liria et al. in their publication [35]. This included postural treatment, passive physical therapy for the lower limb, cryotherapy for an hour three times a day (once after passive mobilization), quadriceps stretching and strengthening exercises, active knee and ankle flexion–extension workouts without resistance, flexion–extension from the sitting position, isotonic exercises, facilitating positional changes from lying to sitting and sitting-to-standing and exercises (short distances) [34]. Transfer exercises, gait training, and stair climbing were all functional exercises.

Krastanova et al. employed a postoperative recovery program following total hip replacement that included breathing exercises that were correlated with the type of exercise (isometric, isotonic), including bilateral lower-limb exercises; analytical gymnastics for paravertebral, abdominal, and upper limb muscles; concentrated isotonic exercises to maintain and improve gluteal muscle strength; exercises that increase range of motion in the hip and knee joints using treadmills and gym equipment; analytical exercises for the primary movements and combined movements of the ankle joint using equipment; and novel methods for proprioceptive neuromuscular facilitation (PNMF). Exercises for balance and postural stability, postisometric relaxation (PIR) for the triceps, assisted stretching to the neutral Thomas test position to prevent flexion contractures and adhesions in the hip joint, gait control, and, when necessary, gait correction using means and aids depending on the stage of recovery are all recommended [35].

The scientific data supporting the best practices for patients receiving ATS need to be standardized in order to facilitate functional rehabilitation [36]. Resistance training has also been shown to boost muscle strength, which is necessary for most everyday activities and ought to be one of the recovery objectives following ATS. The effectiveness of these workouts relies on how many sets and repetitions are completed, as the authors point out. Thus, it is said that recommending 3 to 5 sets of 8 to 10 repetitions for the quadriceps muscle will increase muscle strength.

A study by Umpierres et al. is cited, which showed that 3 sets of 12 repetitions increased the muscle strength of the knee flexors and extensors, as well as the hip abductors, adductors, and rotators [37]. Both healthy people and people who have had total hip arthroplasty are likely to benefit more from protocols consisting of two to three sets of eight to twelve repetitions. There are several studies that show how isometric exercises work very well for increasing the range of motion (ROM) of the hips in ATS patients in terms of flexion, extension, abduction, and internal and external rotation [36,38].

Patients’ self-reported functional improvement in relation to disability, decreases in pain intensity, quality of life, walking speed, muscle strength, and hip joint range of motion were the main advantages highlighted in the majority of the research.

Another significant factor is that a growing proportion of patients are of working age due to a drop in the average age of patients needing this type of intervention [37]. The demands of this shifting generation are no longer being met by conventional methods of ATS recovery.

There is a shortage of strategies for treating preoperatively developed musculoskeletal dysfunctions, myofascial chains, compensatory adjustments, and program adaption to surgical approach, according to the early conclusions and questionnaire analysis.

In this regard, it is suggested to create an intervention model that enables the formulation of practical plans for the implementation and assessment of an ideal recovery program for patients enrolled in recovery programs following PT. It is necessary to evaluate one’s own hypotheses about the prediction of customised recovery plans for patients after total hip arthroplasty.

The shortcomings of the post-AT rehabilitation protocols mentioned in this chapter can be addressed by developing and evaluating a patient-centered, customized therapeutic protocol with content catered to the deficits commonly observed following AT, including the modification of a protocol based on the surgical approach.

While this study did not employ robotic or technological devices as part of the rehabilitation protocol, we acknowledge the growing importance of robot-assisted rehabilitation in the field of orthopedic recovery, including post-THA. Robotic and sensor-based systems offer enhanced precision, real-time feedback, and the potential for individualized, adaptive therapy programs [39]. Studies have shown that such technologies may improve motor control, gait symmetry, and patient motivation during recovery [39]. Although these approaches were beyond the scope of the present study, we acknowledge their clinical relevance and suggest that future research explore the comparative effectiveness of traditional versus technology-assisted rehabilitation protocols in optimizing functional outcomes following THA.

The main benefits reported in most studies were patient self-reported functional improvement in relation to disability, reduction in pain intensity, improvement in quality of life, walking speed, muscle strength, and range of motion in the hip joint. A sophisticated treatment plan should be created for ATS patients with the goal of helping them restore vital abilities like stability, mobility, muscle strength and resistance, motor control, coordination, and balance by adjusting the workouts in this program to target regaining joint mobility, bolstering the hip stabilizer muscles’ strength, battling proprioceptive and balance issues, and retraining the walking gait.

While this study demonstrates valuable insights into using muscle deflection as an indicator of rehabilitation quality after total hip arthroplasty, it also has several limitations. The sample size was relatively small, and patients were selected from only two hospitals, which may limit the generalizability of the findings. Moreover, the lack of randomization and potential variability in patient adherence to the rehabilitation program could introduce bias. The study’s relatively short follow-up period (eight weeks) may not capture long-term outcomes or complications. Despite these limitations, the findings have important implications for clinical practice: incorporating objective measures like muscle deflection into standard rehabilitation protocols could enhance patient monitoring and allow for more personalized therapy adjustments. Future research should include larger, randomized controlled trials with extended follow-up periods to validate these results, assess long-term benefits, and explore how different surgical approaches or patient demographics might influence rehabilitation strategies. Standardizing recovery protocols and integrating proprioception and balance training early in rehabilitation are also recommended to optimize functional outcomes post-THA.

## 5. Conclusions

Our study demonstrates how important a physiotherapist’s efforts are to a patient’s successful recovery from total hip arthroplasty. As a consequence of the planned physiotherapy treatment regimen used in preventive checks, patients who had THA demonstrated functional improvement. According to the study’s findings, this therapy helped patients recover, lessened discomfort, and enhanced their quality of life by enabling them to resume their regular lives.

## Figures and Tables

**Table 1:** Variables and instruments that are measured.

Variable	Measuring Instrument	Measurements	Variable Category
Range of motion of the operated hip joint (hip ROM in the sagittal, frontal, and transverse planes)	Dynamometer	Initial/Final	Dependent
Passive movement	Dynamometer	Initial/Final	Dependent
Muscle strength of the hip abductors and flexors in the operated leg	Dynamometer	Initial/Final	Dependent

**Table 2:** Right hip abduction standing after 2 weeks.

AROM	20.96°
PROM	23.03°
Angle difference	2.07°
Percentage difference	9.41%

**Table 3:** Right hip extension standing after 2 weeks.

AROM	16.89°
PROM	23.21°
Angle difference	6.32°
Percentage difference	31.52%

**Table 4:** Right hip flexion standing after 2 weeks.

AROM	24.35°
PROM	28.82°
Angle difference	4.47°
Percentage difference	16.81%

**Table 5:** Right hip abduction standing after 4 weeks.

AROM	24.03°
PROM	31.66°
Angle difference	7.63°
Percentage difference	27.40%

**Table 6:** Right hip extension standing after 4 weeks.

AROM	21.67°
PROM	35.23°
Angle difference	13.56°
Percentage difference	47.66%

**Table 7:** Right hip flexion standing after 4 weeks.

AROM	27.03°
PROM	29.54°
Angle difference	2.51°
Percentage difference	8.87%

**Table 8:** Right hip abduction standing after 6 weeks.

AROM	22.99°
PROM	30.26°
Angle difference	7.27°
Percentage difference	27.31%

**Table 9:** Right hip extension standing after 6 weeks.

AROM	27.43°
PROM	41.83°
Angle difference	14.40°
Percentage difference	41.58%

**Table 10:** Right hip flexion standing after 6 weeks.

AROM	39.51°
PROM	43.65°
Angle difference	4.14°
Percentage difference	9.96%

**Table 11:** Right hip abduction standing after 8 weeks.

AROM	33.74°
PROM	44.69°
Angle difference	10.95°
Percentage difference	27.92%

**Table 12:** Right hip extension standing after 8 weeks.

AROM	38.74°
PROM	47.29°
Angle difference	8.55°
Percentage difference	19.88%

**Table 13:** Right hip flexion standing after 8 weeks.

AROM	43.61°
PROM	55.37°
Angle difference	11.76°
Percentage difference	23.76%

**Table 14:** Experimental data acquisition of deflection in abduction, extension, and flexion during a period of 2, 4, 6, and 8 weeks after the THA recovery exercises.

No.	Period (Weeks)	Abduction				Extension				Flexion			
		AROM	PROM	Angle Difference	Percentage Difference %	AROM	PROM	Angle Difference	Percentage Difference %	AROM	PROM	Angle Difference	Percentage Difference %
1	2	20.96°	23.03°	2.07°	9.41	16.69°	23.21°	6.63°	31.52	24.35°	28.82°	4.47°	16.47
2	4	24.03°	31.66°	7.63°	27.4	21.67°	35.23°	13.56°	47.66	27.03°	29.54°	2.51°	8.87
3	6	22.99°	30.26°	7.27°	27.31	27.43°	41.83°	14.40°	41.58	39.51°	43.65°	4.14°	9.96
4	8	33.74°	44.69°	10.95°	27.92	38.74°	47.29°	8.55°	19.98	43.61°	55.37°	11.76°	23.76
Average value		25.63°	32.41°	6.98	23.01	26.18°	36.89°	10.71°	35.19	33.63°	39.35°	5.72°	14.77
8–6 growth		1.47°	1.47°	1.51°	1.02	1.41°	1.13°	0.59°	0.48	1.10°	1.26°	2.84°	2.38
8–4 growth		1.40°	1.41°	1.44°	1.01	1.79°	1.34°	0.63°	0.42	1.06°	1.87°	4.68°	2.68
8–2 growth		1.61°	1.94°	5.28°	2.97	2.29°	2.04°	1.29°	0.63	1.79°	1.92°	2.63°	1.44
Average value		1.49°	1.61	2.74°	1.66	1.83°	1.5°	0.84°	0.51	1.32°	1.68°	3.38°	2.16

**Table 15:** The results of the WOMAC tests.

WOMAC Condition of Patients	Before Surgery (THA)		After 8 Weeks of Recovery Exercises		Mean Percentage Difference %
	Mean	Std. Deviation	Mean	Std. Deviation	
Pain	3.16	0.601	1.20	0.501	37.97
Physical difficulties	3.12	0.585	1.00	0.704	32.05
Joint stiffness	3.27	0.884	0.50	0.605	15.29

## Data Availability

The original contributions presented in this study are included in the article. Further inquiries can be directed to the corresponding authors.
